# Comparison of platelet count results on the Sysmex XN between citrate or MgSO_4_ and K2 EDTA anticoagulants

**DOI:** 10.1111/ijlh.13966

**Published:** 2022-09-12

**Authors:** Michel Soulard, Pascale Croix, Patrick Cohen

**Affiliations:** ^1^ Biogroup Hematology Laboratory La Chocolaterie Levallois Perret France; ^2^ Division of Laboratory Medicine, Diagnostic Department Geneva University Hospitals and Faculty of Medicine Geneva Switzerland

**Keywords:** citrate, ethylene diamine tetracetic acid, magnesium sulphate, platelet, Sysmex

## Abstract

**Introduction:**

The aim of this study performed on Sysmex XN is to compare platelet values on citrate and MgSO_4_ (TBX) in patients with K2EDTA‐induced platelet clusters and to identify platelet biases of these matrices compared to K2EDTA.

**Methods:**

Sixty patients with K2EDTA‐induced platelet clusters were re‐sampled with K2EDTA, citrate and TBX. Platelet results were then compared, and smears were analysed for clumping. Platelet results from 120 patients without K2EDTA‐induced platelet clusters were compared between K2 EDTA, citrate, and MgSO_4_ with impedance and fluorescence modes. Biases from regressions were analysed.

**Results:**

Out of the 60 patients with K2EDTA‐induced platelet clusters, none showed platelet clusters with MgSO_4_ whereas 50% still showed clusters with citrate. Among those without platelet clusters on citrate, the mean relative difference between (citrate‐ MgSO_4_)/MgSO_4_ was −12.7% in impedance and −9.8% in fluorescence. Among the 120 patients without K2EDTA‐induced platelet clusters, in fluorescence the mean relative bias with respect to K2EDTA was −2.06% for MgSO_4_ and −10.3% for Citrate. For the MgSO_4_ versus K2 EDTA regressions, the maximum absolute values of the 95% CI of the relative biases at 150 × 10^9^/L (5.45%) and 450 × 10^9^/L (3.56%) were below the desirable analytical objectives of the EFLM.

**Conclusion:**

In patients with K2EDTA‐induced platelet clusters, MgSO_4_ is preferable to citrate. MgSO_4_ provides a bias with XN in fluorescence when compared to EDTA which is within analytical tolerance.

## 1 INTRODUCTION

EDTA dependent pseudothrombocytopenia (PTP) is a problem encountered relatively frequently in daily routine laboratory activity, as the prevalence of K2 EDTA‐induced PTP ranges from 0.03% to 0.27% in the general population.^1^, ^2^, ^3^ PTP represents a challenge because of the risk of delivering an erroneous result of the platelet count, as overlooking a severe thrombocytopenia can have serious consequences for the patient. It is therefore essential to obtain reliable platelet counts in order to make clinically important decisions with confidence.

PTP is characterized by the in vitro agglutination of platelets in the presence of EDTA leading to a falsely lowered platelet count. PTP is confirmed by observation of the May Grunwald Giemsa (MGG) stained blood smear for the presence of platelet clusters and by the disappearance of these platelet clusters using an anticoagulant other than EDTA.

PTP is a temperature and time dependent phenomenon.^4^, ^5^, ^6^ It is related to the existence of predominantly IgG autoantibodies (IgG1) to cryptic epitopes of GpIIbIIIa unmasked by EDTA calcium chelation.^4^, ^5^

To prevent platelet agglutination in vitro, different anticoagulants can be used. Sodium citrate is the most widely used. It is recommended to perform the platelet count within a maximum of 3 h after collection.^5^

Several publications recommend the use of MgSO_4_ for platelet counts in EDTA‐induced PTP.^7^, ^8^ The anticoagulant effect of MgSO_4_ results on the one hand from the inhibition of fibrinogen binding to the GpIIbIIIa complex as well as the inhibition of thromboxane A2 formation, and on the other hand from the formation of cAMP, which blocks platelet activation by inhibiting the mobilization of intra‐tubular calcium.^9^

The Sysmex XN series automated system allows platelets to be counted in two different ways. The impedance after hydrodynamic focalization produces a histogram limited by two flexible thresholds located between 2 and 6 × 10^−15^ L for the lower threshold and between 12 and 30 × 10^−15^ L for the upper threshold. In addition, the use of a method based on the combination of optics and fluorescence allows better discrimination of platelets from leukocyte cytoplasm fragments and improves the accuracy of counting in thrombocytopenic patients with results directly comparable to the reference method CD41/CD61 in flow cytometry.^10^, ^11^, ^12^, ^13^

Previous studies have shown that on the XE 5000 analyser, MgSO_4_ underestimated platelet counts in impedance compared to K3 EDTA but produced similar results in fluorescence using polymethine.^7^, ^8^

The objectives of this study performed on the Sysmex XN analyser were:

‐ In patients with K2EDTA‐induced platelet clusters, to compare the platelet count results obtained when using sodium citrate and MgSO_4_.

‐ In patients without K2EDTA‐induced platelet clusters, to evaluate the regressions and biases at decision thresholds between platelet count results obtained with either sodium citrate or magnesium sulphate and those obtained with K2EDTA using impedance or fluorescence with oxazine.

## 2 MATERIAL AND METHODS

### 2.1 Patients

The patients come from the laboratory's patient base.

We collected samples from 60 patients (33 males, 27 females, median age 67 years, min 19 years, max 92 years) with initial platelet clusters on K2EDTA without fibrin flakes. These samples were collected using 3 tubes and in the following order: first K2EDTA, 7.2 mg, 4 ml, BD Vacutainer; then Sodium Citrate 0.109 M, 2.7 ml, BD Vacutainer; then MgSO_4_, 33.8 μmol, 2.7 ml, Thromboexact (TBX) Sarstedt.

One hundred and twenty patients (65 males, 55 females, median 66 years, min 18 years, max 95 years) without platelet clusters on the blood smear, were sampled for platelet count. For each patient, we collected 1 K2EDTA tube, 1 Sodium Citrate tube and 1 TBX tube. These patients were chosen with a K2EDTA mean corpuscular volume between 75 × 10^−15^ L and 106 × 10^−15^ L, median 91.8 × 10^−15^ L, and leucocytes between 1.42 × 10^9^ /L and 25.53 × 10^9^/L, median 7.21 × 10^9^/L, without cytoplasmic fragments of nucleated cells on the May Grunwald Giemsa (MGG) stained smear. In addition, no images suggestive of cryoglobulin were observed on the smears. One patient had platelet aggregates on the K2EDTA blood smear that were grouped by 2 or 3 at most on 4 out of 10 fields. This patient, whose largest clusters were less than 5 platelets, was not excluded from the study. On K2EDTA, this patient had 213 × 10^9^/L platelets in impedance and 249 × 10^9^/L platelets in fluorescence; and on MgSO4 he had 259 × 10^9^/L platelets in impedance and 258 × 10^9^/L platelets in fluorescence.

This study was approved by the Ethics Committee (Ile de France IV) (2021‐A02741‐40) and was conducted according to the Declaration of Helsinki. All patients gave written informed consent for participation in the study.

### 2.2 Sample processing

Samples were transported at 15–25°C and analysed within a maximum of 3 h after collection. Platelets from the 60 patients with platelet clusters were analysed using an XN 9000 (XN10 version 22.16). Platelets from the 120 patients without platelet clusters were analysed in replicate using the same XN3000 (XN10 version 22.16) in impedance and fluorescence with oxazine. None of the 120 samples showed alarms on the XN suggesting the presence of platelet clusters.

During the study period, three levels of internal quality control materials were routinely evaluated for the XN 3000 and XN 9000 and all the required maintenance procedures were performed in accordance with the Sysmex's manual. The laboratory also obtained acceptable results on external quality control samples.

Platelet counts on citrate were corrected by multiplying the raw result by the ratio of haemoglobin levels on K2EDTA to citrate. Platelet clusters were detected by staining smears with MGG on K2EDTA, citrate, and TBX tubes from patients. When observing 10 fields under the microscope (50X, 10X), the presence of platelet clusters was characterized by the observation on each of at least four fields of platelets attached by five. Blood smears were read under a Zeiss light microscope (Primo Star) by a laboratory technician and verified by a senior haematopathologist.

### 2.3 Analysis of statistical results

The results of the platelet count on citrate and MgSO4 were compared for each mode with the result obtained on K2EDTA. Three regressions were analysed: least squares (O.L.S), Deming, and Passing‐Bablok (PB). For Deming, the ratio of analytical variances was calculated according to Cornbleet and Gochman.^14^ For each regression, the slope, intercept, R2 coefficient, and biases at the 150 × 10^9^/L and 450 × 10^9^/L thresholds were evaluated. The 95% CI of the intercept, slope, and biases were calculated for O.L.S according to Clinical and Laboratory Standards Institute^15^ with Excel 2010, for Deming according to Jacknife^16^, ^17^ with Excel 2010, and for PB according to Bootstrap with MedCalc® Statistical Software version 20.011 (MedCalc Software Ltd, Ostend, Belgium). Relative biases were compared to the desirable EFLM^18^ (European Federation of Clinical Chemistry and Laboratory Medicine Biological variation) biases and the maximum bounds of the 95%CI of the biases were compared to the reference data.^19^

The Shapiro–Wilk test was used to analyse the normality of the distribution of results. Non‐normally distributed data was expressed as medians and interquartile ranges (IQR).

Means were compared using Student's *t*‐test when the difference in values was normally distributed, otherwise the Wilcoxon test was performed. Medians were compared with the Friedman test. The significance level is *p* < 0.05.

## 3 RESULTS

### 3.1 Comparison of platelet results with citrate or MgSO_4_ in patients with K2EDTA induced platelet clusters

Platelet counts were performed in impedance mode in all the 60 patients sampled on K2EDTA, Citrate and MgSO_4_.

On K2EDTA, the median was 67 × 10^9^/L, on citrate the mean was 148.8 × 10^9^/L, on MgSO4 the mean was 209.6 × 10^9^/L.

All patients had platelet clusters on K2EDTA. Thirty of them still showed clusters on citrate (50%) and none showed clusters on MgSO4. All platelet results obtained with MgSO4 were superior to those of K2EDTA and citrate. It should be noted that eight patients had lower platelet results on citrate than on K2EDTA (Table 1).

**TABLE 1:** Comparison of platelet count results (10^9^/L) obtained on the XN impedance analyser using the anticoagulants citrate and MgSO_4_ in 60 patients with K2EDTA‐induced platelet clusters

Patients	K2EDTA	Citrate	MgSO4	Patients	K2EDTA	Citrate	MgSO_4_
1	**170**	**108**	215	31	**40**	218	242
2	**32**	**61**	134	32	**118**	208	237
3	**17**	**99**	245	33	**73**	96	135
4	**62**	**82**	137	34	**30**	101	140
5	**64**	**134**	195	35	**52**	132	159
6	**47**	**154**	241	36	**47**	292	324
7	**183**	**83**	230	37	**80**	116	142
8	**36**	**101**	151	38	**55**	180	211
9	**89**	**109**	193	39	**249**	294	334
10	**81**	**218**	281	40	**72**	236	266
11	**70**	**125**	164	41	**84**	199	229
12	**44**	**163**	278	42	**136**	232	254
13	**147**	**140**	209	43	**38**	247	275
14	**39**	**100**	143	44	**61**	182	200
15	**27**	**12**	209	45	**38**	94	119
16	**119**	**114**	152	46	**25**	188	202
17	**71**	**234**	358	47	**118**	130	164
18	**66**	**113**	212	48	**86**	202	242
19	**134**	**152**	247	49	**102**	266	282
20	**15**	**108**	173	50	**63**	128	148
21	**41**	**108**	178	51	**58**	108	126
22	**19**	**40**	109	52	**99**	272	297
23	**84**	**187**	266	53	**104**	197	225
24	**35**	**147**	191	54	**24**	216	224
25	**86**	**14**	232	55	**85**	133	148
26	**114**	**68**	290	56	**137**	194	228
27	**238**	**134**	298	57	**100**	178	195
28	**31**	**42**	201	58	**49**	150	164
29	**113**	**196**	272	59	**68**	143	148
30	**64**	**93**	138	60	**66**	159	173

*Note*: Platelet count results with platelet clusters are shown in bold.

Of the 60 patients whose platelet results were associated with the presence of platelet clusters, 56 had EDTA‐induced pseudothrombopenia. With citrate, 33 out of the 56 patients had platelet counts below 150 × 10^9^/L, 10 of which were confirmed by the absence of platelet clusters on the smear. In contrast, with MgSO_4_, 13 patients had platelet counts below 150 × 10^9^/L with no clumps on the smear. For patient 47, the thrombocytopenia diagnosed with citrate disappeared with MgSO_4_.

Among the 30 patients without platelet clusters on citrate, the mean of the relative differences in impedance analysed platelets between Citrate and MgSO_4_ versus MgSO_4_ for these patients was −12.7%, 95% CI (−10.4% to −14.9%).

Out of the previous 60 patients, 30 were analysed by fluorescence (eight with clusters on citrate and 22 without clusters).

The median was 98.5 × 10^9^/L on K2EDTA, while the medians were 207.6 × 10^9^/L on citrate and 255.3 × 10^9^/L on MgSO_4_.

Among the 22 patients without platelet clusters on citrate, the mean relative difference in platelets between Citrate and MgSO_4_ versus MgSO_4_ was −9.8%, 95%CI (−5.7% to −13.9%) in fluorescence −11.3%, 95%CI (−7.9% to −14.7%) in impedance.

For these 22 patients the mean relative difference in platelets between fluorescence and impedance versus impedance was 12.17%, 95% CI (7.03%–17.31%) on citrate and 10.97%, 95% CI (3.65 to 18.28) on MgSO_4_.

These results suggested that in patients with EDTA‐induced platelet clusters, MgSO_4_ was more effective than citrate to perform platelet counts. Furthermore, on XN with MgSO_4_ the platelet results obtained with fluorescence were about 10% higher than those obtained with impedance.

### 3.2 Comparison of platelet results between MgSO_4_ and K2EDTA in patients without K2EDTA induced platelet clusters

One hundred and twenty patients were sampled with K2 EDTA and MgSO_4_. Platelet counts were performed on a single XN using impedance and fluorescence modes.

For K2EDTA the range of values was 18–767 × 10^9^/L in impedance and 15–801 × 10^9^/L in fluorescence.

For all these patients the mean relative bias of the platelet results between fluorescence and impedance compared to impedance was −0.45% 95%CI (−1.85 to 0.95) with K2EDTA and 9.45% 95%CI (7.81–11.09) with MgSO_4_.

Therefore, the results of platelet count in the fluorescence correlated better with K2EDTA than with MgSO_4_ in impedance.

Comparison of platelet count results in fluorescence mode between MgSO_4_ and K2EDTA versus K2EDTA showed a mean relative bias of −2.06% 95%CI (−3.41 to −0.71).

**FIGURE 1:**
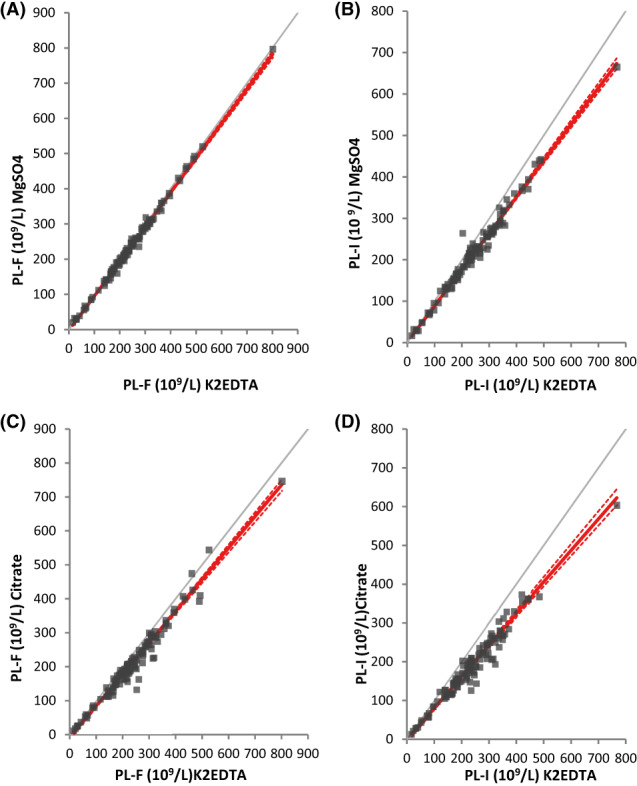
Comparison using Passing‐Bablok regression of platelet results from 120 patients without platelet clumps between magnesium sulfate and K2EDTA (A, B) and between sodium citrate and EDTA K2 (C, D) in fluorescence (A, C), and impedance (B, D) modes. Regression lines are presented with solid red lines, 95%CI lines are presented with dashed red lines, and identity lines are presented with solid grey lines. PL‐I, platelets measured on the XN analyser in impedance mode; PL‐F, platelets measured on the XN analyser in fluorescence mode; Citrate, sodium citrate; magnesium sulfate, MgSO_4_.

In fluorescence the MgSO_4_ versus K2EDTA regressions showed correlation coefficients greater than 0.995. The slope of the Deming regression was not statistically different from 1. At the decision threshold of 150 × 10^9^/L, the average bias was between 4.6 × 10^9^/L and 5.4 × 10^9^/L and the maximums from 95% CI were between 6.7 × 10^9^/L and 8.2 × 10^9^/L. At the 150 × 10^9^/L and 450 × 10^9^/L bounds, the mean relative bias and the maximum relative bias derived from the 95%CI were lower than the specifications of the EFLM^18^ and Buoro et al.,^19^ respectively (Table 2 Figure 1A).

**TABLE 2:** Comparison of platelet results obtained between magnesium sulfate (MgSO_4_) and K2EDTA (EDTA) in 120 patients without platelet clumps on XN analyser with impedance or fluorescence mode using least squares, Deming and Passing‐Bablok regressions

Méthod	Comparison	X‐max/X‐min (10^9^/L)	Regression	Slope 95CI	Intercept 95CI	C.C.	Bias (%) Xc = 150 10^9^ /L	Max Bias (%) Xc = 150 10^9^/L	Bias (%) Xc = 450 10^9^/L	Max Bias (%) Xc = 450 10^9^/L
Fluo	Y = MgSO_4_ versus X = EDTA	801/15	OLS	0.984 (0.972 to 0.997)	−3.037 (−6.415 to 0.341)	0.997	**3**.**58**	**5**.**45**	**2**.**23**	**3**.**05**
Fluo	Y = MgSO_4_ versus X = EDTA	801/15	Deming	0.988 (0.974 to 1.002)	−3.907 (−7.318 to −0.497)	0.997	**3**.**81**	**5**.**00**	**2**.**08**	**2**.**84**
Fluo	Y = MgSO_4_ versus X = EDTA	801/15	PB	0.978 (0.962 to 0.992)	−1.374 (−4.937 to 1.555)	0.995	**3**.**08**	**4**.**45**	**2**.**47**	**3**.**56**
Imp	Y = MgSO_4_ versus X = EDTA	767/18	OLS	0.870 (0.848 to 0.893)	3.7 05 (−2.261 to 9.671)	0.990	10.48	13.73	12.13	13.58
Imp	Y = MgSO_4_ versus X = EDTA	767/18	Deming	0.878 (0.860 to 0.897)	1.792 (−2.420 to 6.004)	0.990	10.96	12.73	11.75	13
Imp	Y = MgSO_4_ versus X = EDTA	767/18	PB	0.876 (0.856 to 0.896)	1.874 (−1.740 to 6.152)	0.979	11.19	12.7	12.02	13.3
Fluo Imp	Y = MgSO_4_ F versus X = EDTA I	767/18	OLS	1.008 (0.980 to 1.036)	−7.664 (−15.122 to −0.206)	0.988	**4**.**29**	8.35	**0**.**88**	**2**.**7**
Fluo Imp	Y = MgSO_4_ F versus X = EDTA I	767/18	Deming	1027 (0.995 to 1059)	−12.165 (−19.538 to −4.791)	0.988	5.41	8.53	**0**	**1**.**89**
Fluo Imp	Y = MgSO_4_ F versus X = EDTA I	767/18	PB	0.997 (0.960 to 1026)	−4.624 (−12.488 to 3.089)	0.981	**3**.**41**	**6**.**03**	**1**.**35**	**3**.**55**

*Note*: Shown in bold are the mean relative biases that are less than the desirable specifications of the EFLM platelet bias and the maximum relative biases that are less than the 95% confidence interval limits of the platelet bias according to Buoro et al.^19^

Abbreviations: Bias (%) Xc, absolute value of the relative bias measured at medical decision level Xc; C.C., correlation coefficient; Deming, Deming regression; F, Fluo, Fluorescence; I, Imp, Impedance, OLS, ordinary least squares regression; Max Bias (%), absolute value of the maximum of the 95% confidence interval of the relative bias at medical decision level Xc; PB, Passing‐Bablok regression.

By way of comparison, at a threshold of 150 × 10^9^/L, in impedance, the MgSO_4_ versus K2EDTA regressions generated mean biases between 15.7 × 10^9^/L and 16.8 × 10^9^/L and maximum biases between 19 × 10^9^/L and 20.6 × 10^9^/L (Table 2, Figure 1B).

Regression analysis of Passing Bablok (PB), MgSO_4_ in fluorescence versus K2EDTA in impedance, showed a slope that was not statistically different from 1 and a mean bias at 150 × 10^9^/L of 5.1 × 10^9^/L.

As a result, platelet counts on MgSO_4_ analysed in fluorescence correlate with platelet counts on K2EDTA analysed in impedance and were similar to those obtained with K2EDTA in fluorescence.

### 3.3 Comparison of platelet results between Citrate and K2EDTA in patients without K2EDTA induced platelet clusters

For the 120 patients, the mean relative bias of platelets on citrate between fluorescence and impedance compared to impedance was 9.49% 95% CI (7.60%–11.39%).

The results of platelets collected on citrate were always lower than those of platelets collected on K2 EDTA regardless of the mode of analysis.

For the 120 patients, the median platelet (citrate‐K2EDTA)/K2EDTA result ratio was in impedance −18.7% IQR (−24.6%; −14.5%) and in fluorescence −10.5% IQR (−15.5%; −7%).

Analysis of the results of the platelet regressions between citrate and K2EDTA showed correlation coefficients ranging from 0.942 to 0.96 in impedance and from 0.953 to 0.977 in fluorescence. The mean biases of the citrate versus K2EDTA regressions at the decision threshold of 150 × 10^9^/L ranged from 15.4 × 10^9^/L to 21.5 × 10^9^/L in fluorescence and from 27.9 × 10^9^/L to 30.4 × 10^9^/L in impedance (Table 3, Figure 1C, D).

**TABLE 3:** Comparison of platelet results obtained between sodium citrate (Cit.) and EDTAK2 (EDTA) in 120 patients without platelet clumps on XN analyser with impedance or fluorescence mode using least squares, Deming and Passing‐Bablok regressions

Méthod	Comparaison	X max ‐X min (10^9^/L)	Regression	Slope 95CI	Intercept 95CI	C.C.	Bias (%) Xc = 150 10^9^ /L	Max Bias (%) Xc = 150 10^9^/L	Bias (%) Xc = 450 10^9^/L	Max Bias (%) Xc = 450 10^9^/L
Imp	Y = Cit. versus X = EDTA	767–18	OLS	0.793 (0.757–0.829)	0.635 (−8.817 to 10.087	0.942	20.30	25.44	20.58	22.88
Imp	Y = Cit. versus X = EDTA	767–18	Deming	0.870 (0.823–0.916)	4.853 (−6.164 to 15.870)	0.956	19.16	21.9	19.8	22.2
Imp	Y = Cit. versus X = EDTA	767–18	PB	0.812 (0.782–0.847)	0.318 (−7.171 to 4.518	0.96	18.61	21.39	18.76	21.03
Fluo	Y = Cit. versus X = EDTA	801–15	OLS	0.920 (0.833–0.958)	−9.624 (−19.532 to 0.283)	0.953	14.31	19.82	10.06	14.28
Fluo	Y = Cit. versus X = EDTA	801–15	Deming	0.935 (0.889–0.980)	−10.595 (−21.485 to 0.294)	0.977	13.6	17.12	8.89	11.4
Fluo	Y = Cit. versus X = EDTA	801–15	PB	0.924 (0.894–0.948)	−3.94 (‐11.458 to −0.734)	0.97	10.24	13.11	8.49	10.41

Abbreviations: Bias (%) Xc, absolute value of the relative bias measured at medical decision level Xc; C.C., correlation coefficient; Deming, Deming regression; Fluo, Fluorescence; Imp, Impedance, OLS, ordinary least squares regression; Max Bias (%), absolute value of the maximum of the 95% confidence interval of the relative bias at medical decision level Xc; PB, Passing‐Bablok regression.

Although the regression results for citrate versus K2EDTA platelets were slightly better in the fluorescence mode than in the impedance mode, at thresholds of 150 × 10^9^/L, the mean relative biases and their 95%CI exceeded the analytical specifications of the EFLM^18^ and Buoro et al.^19^

### 3.4 Mean platelet volume

Among the 113 patients for whom platelet volume was available, a comparison of results between K2EDTA, citrate, and MgSO_4_ matrices was performed.

Medians of platelet volume with interquartile ranges (IQR) were 10.3 × 10^−15^/L (9.8; 10.94), 9.75 × 10^−15^/L (9.3; 10.40), and 9.50 × 10^−15^/L (9.1; 10.08) for K2EDTA, citrate, and MgSO4 respectively. They differed significantly (Friedman test *p* < 0.0001).

The mean relative bias between citrate and EDTA K2 was −5.48%, 95%CI (−6.207 to −4.748) while the mean relative bias between TBX and EDTA K2 was −7.4%, 95%CI (−8.117 to −6.682).

The bias of citrate over K2EDTA from the Passing Bablok (PB) regression was −0.6 × 10^−15^/L 95%CI (−0.71 to −0.49) for MPV K2EDTA = 9 × 10^−15^/L and was −0.6 × 10^−15^/L 95%CI (−0.73 to −0.39) for MPV K2EDTA = 12 × 10^−15^/L.

The bias of MgSO4 vs EDTA from PB regression was −0.67 × 10^−15^/L 95CI (−0.8 to −0.56) for MPV K2EDTA = 9 × 10^−15^/L and was −0.95 × 10^−15^/L, 95CI (−1.12 to −0.79) for MPV EDTA = 12 × 10^−15^/L.

At the 9 × 10^−15^/L and 12 × 10^−15^/L thresholds, the absolute value of the relative biases obtained between MgSO4 and K2EDTA and those obtained between citrate and K2EDTA exceeded the relative bias of 1.9% (95%CI: 1.4%–2.7%) established by Buoro et al.^19^

## 4 DISCUSSION

PTP is a relatively common in vitro phenomenon which leads to falsely low platelet counts determined automatically. Usually, the thrombocytopenia is noticed accidentally without a corresponding tendency to bleeding. But if this interference is not recognized, misinterpretation may have dramatic consequences by way of diagnostical and therapeutical mistakes. Besides, in many patients, the use of another matrix such as sodium citrate does not overcome this interference and does not solve the problem. This is the reason why it is important to have a matrix that allows the laboratory to deliver a correct platelet count on which the clinician can base his diagnostic and/or therapeutic attitude.

This study showed that in 60 patients with K2EDTA‐induced clumping, the use of MgSO4 was more effective than citrate in removing platelet clumps. In addition, on XN, fluorescence counting of platelets collected on MgSO4 generated an average relative bias of −2.06% compared to K2EDTA and relative biases at the threshold of 150 × 10^9^/L which, regardless of the regression procedure, remained below the desirable specifications of the EFLM.^18^ On the other hand, the use of citrate in fluorescence or MgSO4 in impedance did not achieve these objectives.

Comparison of the mean values of the platelet ratios (citrate‐ MgSO4)/MgSO4 measured by impedance: −12.7% (30 patients) and −11.3% (22 patients), and fluorescence: −9.8% (22 patients), suggested that irrespective of the mode of analysis, impedance or fluorescence, the results of platelets count on citrate were on average about 10% lower than those on MgSO_4_ measured in the same mode.

Comparison of the mean values of the platelet ratios (fluorescence‐impedance)/impedance obtained on citrate: 12.7% (22 patients) and 9.49% (120 patients), and on MgSO_4_: 10.97% (22 patients) and 9.45% (120 patients), suggested that regardless of the matrix, MgSO_4_ or citrate, the platelet results on one of these matrices were on average about 10% higher on fluorescence than on impedance. In contrast, for EDTA, the platelet fluorescence results were on average about 0.45% lower than the platelet impedance results.

This latter ratio, which reflects the overestimation of platelets in impedance, must be interpreted according to the selection criteria of the 120 patients. First, samples that showed platelet clusters on the smear consisting of at least 5 agglutinated platelets were eliminated. In addition, for red blood cells, only two patients had an K2EDTA MCV of less than 80 × 10^−15^ L: one with an K2EDTA MCV of 75 × 10^−15^ L with platelets at 305 × 10^9^/L in impedance and 296 × 10^9^/L in fluorescence, and the other with an K2EDTA MCV of 77 × 10^−15^ L and platelets at 336 × 10^9^/L in impedance and 301 × 10^9^/L in fluorescence. Finally, for red blood cells, the RDW‐CV ranged from 11.5% to 19% and the fragment alarm was never generated.

Two patients excluded from the study had a deep microcytosis: one with a K2EDTA MCV of 62 × 10^−15^/L had platelets in fluorescence at 161 × 10^9^/L on K2EDTA and 159 × 10^9^/L on MgSO_4_, the other one with a K2EDTA MCV of 55 × 10^−15^/L had platelets at 171 × 10^9^/L on K2EDTA and 168 × 10^9^/L on MgSO_4_. These results suggest that even in the case of microcytosis, platelet fluorescence results on MgSO_4_ correlate well with those obtained on K2EDTA although this needs to be confirmed in a larger patient population.

Since the average relative bias between MgSO_4_ Fluorescence and K2EDTA Fluorescence compared to K2EDTA Fluorescence was low (−2.06%), all platelets detected in fluorescence by K2EDTA were likely to be detected with MgSO_4_. The underestimation of MgSO_4_ platelets analysed by impedance compared to K2EDTA might be related to the reduced volume of platelets collected on MgSO_4_ compared to K2EDTA which would result in the loss of platelets below the low detection limit. The underestimation of citrate platelets analysed by impedance compared to K2EDTA was not explained by the decrease in platelet volume of citrate compared to K2EDTA, as the MPV of citrate was higher than the MPV of MgSO_4_ and therefore the platelet counts in citrate impedance should be higher than those obtained in MgSO_4_.

Conversely, it can be seen that at a threshold of 150 × 10^9^/L in impedance, the mean bias between MgSO_4_ and K2EDTA was 17 × 10^9^/L, whereas it was 26 × 10^9^/L between citrate and K2EDTA.

The mean relative bias in fluorescence between Citrate and K2EDTA compared to K2EDTA (−10.3%) revealed that citrate, unlike MgSO_4_, detected a lower number of platelets in fluorescence compared to K2EDTA. This difference could be explained either by a lower reactivity of the fluorochrome oxazine with platelets in the presence of citrate compared to K2EDTA, or by the fact that some platelets aggregated in the presence of citrate and were excluded from the count due to the large size of the platelet clusters.^20^

In a study of 23 patients, François et al.^21^ showed that with ADVIA, the median platelet count on K3EDTA (255 × 10^9^/L) was higher than on MgSO_4_ (215 × 10^9^/L) and that the median platelet volume on K3EDTA (8.6 × 10^−15^ L) was lower than on MgSO_4_ (10.45 × 10^−15^ L). These results suggest, according to the authors, that the decrease in platelets on MgSO_4_ compared to K3EDTA is related to platelet activation and fragmentation. Our results obtained on XN in oxazine fluorescence and those of Mannuß obtained on XE in optics show much lower average platelet deviations between the 2 matrices and the MPV measured in impedance are higher for K2EDTA or K3EDTA than for MgSO_4_. These discrepancies are related to the ADVIA assay method, which relies on the interaction between the laser beam and the platelets to obtain the volume and refractive index which are then converted to platelet volume and density. The greater increase in MPV over time with K3EDTA compared to MgSO_4_ on ADVIA could result in altered optical properties and a defect in platelet counting.^8^ In contrast, the hypothesis of platelet activation on MgSO_4_ was refuted by the observation that the activation marker CD62 is less expressed on platelets in the presence of MgSO_4_ or citrate compared to EDTA.^7^

In summary, in K2EDTA induced platelet clusters, MgSO_4_ is more effective than citrate in removing platelet clusters. Consequently, in a PTP context, we advocate the use of the MgSO_4_ matrix rather than citrate to overcome this interference and deliver a correct platelet count. In addition, unlike citrate, the use of MgSO_4_ results in a bias on fluorescence XN compared to K2EDTA that is compatible with the desirable analytical specifications of the EFLM.^18^ Further studies will be required to compare the accuracy of platelet results obtained with MgSO_4_ versus K2EDTA and to clarify the stability of platelets collected on MgSO_4_ when analysed in fluorescence with XN.

## AUTHOR CONTRIBUTIONS

Michel Soulard designed the study. Pascale Croix performed the experiments. Michel Soulard, Patrick Cohen analysed the data and wrote the manuscript. All the authors read and approved the content of the manuscript.

## FUNDING INFORMATION

The authors declare that they have not received any funding.

## CONFLICT OF INTEREST

The author declares that there is no conflict of interest.

## ETHICS STATEMENT

This study received the approval of the Ethics Committee (Ile de France IV) (2021‐A02741‐40).

## PATIENT CONSENT STATEMENT

All patients gave written informed consent for participation in the study.

## Data Availability

The data that support the findings of this study are available from the corresponding author upon reasonable request.
